# High throughput sequencing of small RNAs transcriptomes in two *Crassostrea* oysters identifies microRNAs involved in osmotic stress response

**DOI:** 10.1038/srep22687

**Published:** 2016-03-04

**Authors:** Xuelin Zhao, Hong Yu, Lingfeng Kong, Shikai Liu, Qi Li

**Affiliations:** 1 Key Laboratory of Mariculture, Ministry of Education, Ocean University of China, Qingdao 266003, China; 2 The Fish Molecular Genetics and Biotechnology Laboratory, School of Fisheries, Aquaculture, and Aquatic Sciences and Program of Cell and Molecular Biosciences, Auburn University, Auburn, AL 36849, USA

## Abstract

Increasing evidence suggests that microRNAs post-transcriptionally regulate gene expression and are involved in responses to biotic and abiotic stress. However, the role of miRNAs involved in osmotic plasticity remains largely unknown in marine bivalves. In the present study, we performed low salinity challenge with two *Crassostrea* species (*C. gigas* and *C. hongkongensis*), and conducted high-throughput sequencing of four small RNA libraries constructed from the gill tissues. A total of 202 and 87 miRNAs were identified from *C. gigas* and *C. hongkongensis*, respectively. Six miRNAs in *C. gigas* and two in *C. hongkongensis* were differentially expressed in response to osmotic stress. The expression profiles of these eight miRNAs were validated by qRT-PCR. Based on GO enrichment and KEGG pathway analysis, genes associated with microtubule-based process and cellular component movement were enriched in both species. In addition, five miRNA-mRNA interaction pairs that showed opposite expression patterns were identified in the *C. hongkongensis*, Differential expression analysis identified the miRNAs that play important regulatory roles in response to low salinity stress, providing insights into molecular mechanisms that are essential for salinity tolerance in marine bivalves.

MicroRNAs (miRNAs) are a class of endogenous small noncoding RNAs, which regulate gene expression post-transcriptionally in animals and plants^1^. MiRNAs are abundant in animal genomes and have been reported to play primary roles in a broad range of biological and metabolic processes, such as regulation of individual development^2^,^3^, abiotic stress response^4^,^5^, pathogen defense and innate immune response^6^,^7^,^8^. In a previous study, more than 60% of human protein-coding genes are predicted as targets of miRNAs based on *in silico* analysis^9^.

Recent progress in the development of genomic techniques, especially high-throughput sequencing, has greatly facilitated transcriptome analysis of ecologically and economically important animals. Next-generation sequencing based RNA-seq analysis has been widely used to uncover expression patterns under different conditions^10^,^11^. Recently, high-throughput sequencing of small RNA transcriptomes for miRNA discovery and expression profiling has been conducted in numerous model organisms^12^,^13^ and plants^4^,^14^. Next-generation sequencing technologies have also been used for miRNA studies in a number of non-model organisms including several fish species^15^,^16^,^17^,^18^,^19^, echinoderms^20^,^21^,^22^,^23^,^24^, and marine bivalves^7^,^25^,^26^,^27^,^28^,^29^.

*Crassostrea* is one of the most studied bivalve genera due to its worldwide distribution, strong adaptability and high economic importance. The ability to cope with abiotic and biotic stresses is vital to survival of the oysters because of their intertidal inhabiting lifestyle. It has long been an interesting question how the oysters could be survived in response to the environmental stresses involved in the fluctuation of salinity, and air exposure. It is reported that the biogenesis of miRNA, the expression of mRNA targets, and the activities of miRNA-protein complexes were actively regulated under stress conditions^30^. Many studies have been performed to reveal the alterations of mRNA expression^31^,^32^,^33^,^34^,^35^,^36^ and protein expression^37^,^38^,^39^,^40^,^41^ under abiotic and biotic stresses in the oyster^42^, while expression profiling of miRNAs under osmotic stress remains largely unexplored.

The Pacific oyster (*C. gigas*) and the Hongkong oyster (*C. hongkongensis*) are two major *Crassostrea* species along the coasts of China^43^. These two oyster species form distinct fauna assemblages. *C. gigas* can live in the environments with salinity over 20‰^44^, while *C. hongkongensis* inhabits in the estuarine condition with river disemboguing^45^,^46^. Due to the drastic differences in salinity tolerance, the *C. gigas* and *C. hongkongensis* provide an excellent model system to study the roles of miRNAs in adaptation to hypo-osmotic stress.

In this work, we performed high-throughput sequencing of small RNA transcriptomes of gill tissues in *C. gigas* and *C. hongkongensis* after osmotic stress treatment. The gill tissue is a primary interface between the hemolymph or cytoplasm and the external environment in marine molluscs whose osmolality fluctuates widely^47^. Gill is known to be responsive to environmental challenges at the transcript level^35^,^48^ and proteomic level^41^. Based on the recently released genome sequence of the Pacific oyster^49^ and the global expression profile of mRNA transcripts in the gill tissues from our previous study^48^, we identified known and novel miRNAs from the two oysters. The integrated analysis of miRNA and mRNA expression profiles in the two oyster species allowed identification of miRNA-mRNA interaction pairs. Further Gene Ontology and pathway analysis enabled the investigation of their putative biological functions. This work generated genomic resources of miRNAs that are valuable for further studies in the oysters, and provided insights into the roles of miRNAs in response to low salinity stress.

## Results

### High-throughput sequencing of small RNAs

Four small RNA libraries constructed from the two groups of *C. gigas* (one in salinity 25‰, PC; one in salinity 8‰, PT) and two groups of *C. hongkongensis* (one in salinity 25‰, HC; one in salinity 8‰, HT). High-throughput small RNA transcriptome sequencing generated a total of 13,268,157, 14,682,519, 22,288,517, and 21,645,549 raw reads from PC, PT, HC and HT groups, respectively. After removing low quality reads and adaptors, a total of 320,436, 429,972, 464,695 and 572,293 unique clean reads from PC, PT, HC and HT groups, respectively. These unique clean reads were further annotated based on the Pacific oyster genome, and were classified into miRNAs, tRNAs, rRNAs and others by blasting with Rfam and miRBase (Table 1). The reads annotated with other noncoding RNAs rather than miRNAs were excluded for further analysis. The unannotated RNAs were used to identify novel miRNAs.

**Figure 1:**
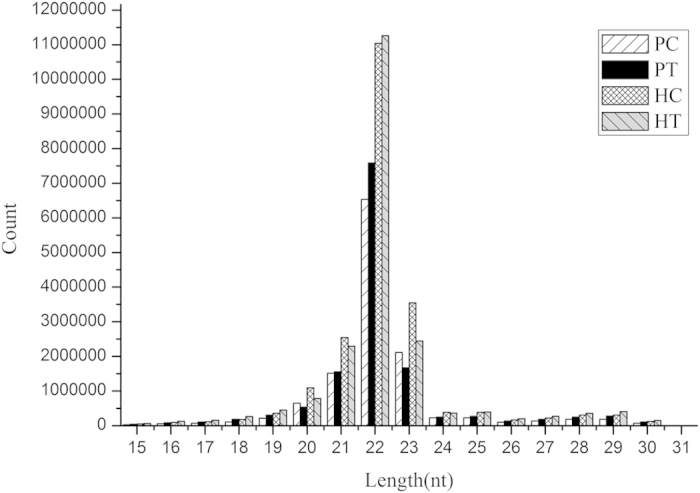
Length distribution of small RNAs in four groups from *C. gigas* and *C. hongkongensis.*

The size distribution of all miRNAs indicated that the majority of miRNAs were with lengths of 21–23 nt long (Fig. 1). The majority of reads were with length of 22 nucleotides in the four libraries miRNAs occupied the major percentages (52.7% for PC, 56.1% for PT, 52.9% for HC, 56.4% for HT) in the four libraries.

### Identification of conserved miRNAs

A total of 137 and 85 conserved miRNAs were identified in the *C. gigas* (PC: 132, PT: 137) and *C. hongkongensis* (HC: 82, HT: 81), respectively. One hundred and seven miRNAs in *C. gigas* and 54 miRNAs in *C. hongkongensis* with read counts greater than 10 were listed in Table S1. A total of 39 miRNAs were identified in both species.

Comparison of the expression of all conserved miRNAs within the two oyster species revealed that miR-10a was expressed with the highest level in each group of both species. The expression of miR-10a was followed by miR-184, miR-184-3p, miR-10b and miR-981 in *C. gigas*, while was followed by miR-981, miR-8, miR-10-5p and miR-2001 in *C. hongkongensis* (Table S1).

### Identification of novel miRNAs

The precursors of several unannotated miRNAs were identified based on the *C. gigas* genome^49^ and *C. hongkongensis* transcriptome^48^. A total of 65 novel miRNAs were identified in the *C. gigas* and two novel miRNAs were identified in the *C. hongkongensis*, respectively (Table S2). Of the 65 novel miRNAs from *C. gigas*, 32 and 37 novel miRNAs were identified in PC and PT groups, respectively, with four novel miRNAs being identified in both groups. In *C. hongkongensis*, only one novel miRNA was identified in both groups and the other one was only identified in the HT group.

### Identification of differentially expressed miRNAs after osmotic stress

Based on the statistical analysis of read counts in the four miRNA libraries, a total of six differentially expressed miRNAs (three up-regulated and three down-regulated miRNAs) between PC and PT groups were identified in the *C. gigas* and two differentially expressed miRNAs (one up-regulated and one down-regulated miRNAs) between HC and HT groups were identified in the *C. hongkongensis* (Table 2). The miR-2353 was identified to be down-regulated in both species.

### Validation of differentiallly expressed miRNAs by qRT-PCR

**Figure 2:**
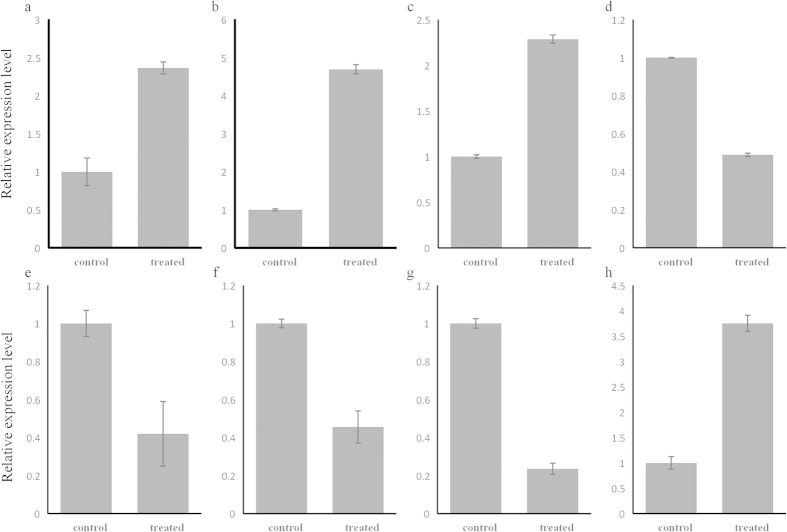
Expression of eight miRNAs determined by qRT-PCR. The eight miRNAs included scaffold43364_10952 (**a**), miR-92-3p (**b**), miR-1984 (**c**), miR-183 (**d**), miR-184-3p (**e**), and miR-2353 (**f**) in *C. gigas* and miR-2353 (**g**) and miR-3205 (**h**) in *C. hongkongensis*. 5S gene was used as an internal control to calibrate the cDNA template for all the samples. Each values were shown as mean ± SD (n = 6).

Those six differentially expressed miRNAs (scaffold43364_10952, cgi-miR-1984, cgi-miR-92, cgi-miR-183, cgi-miR-2353, cgi-miR-184-3p) identified in *C. gigas* and two differentially expressed miRNAs (chk-miR-3205 and chk-miR-2353) identified in the *C. hongkongensis* were validated by quantitative real-time PCR. All of these miRNAs showed a consistent expression pattern with the results from small RNA sequencing (Fig. 2), indicating high reliability of the analysis.

### Prediction and annotation of miRNA target genes

**Figure 3:**
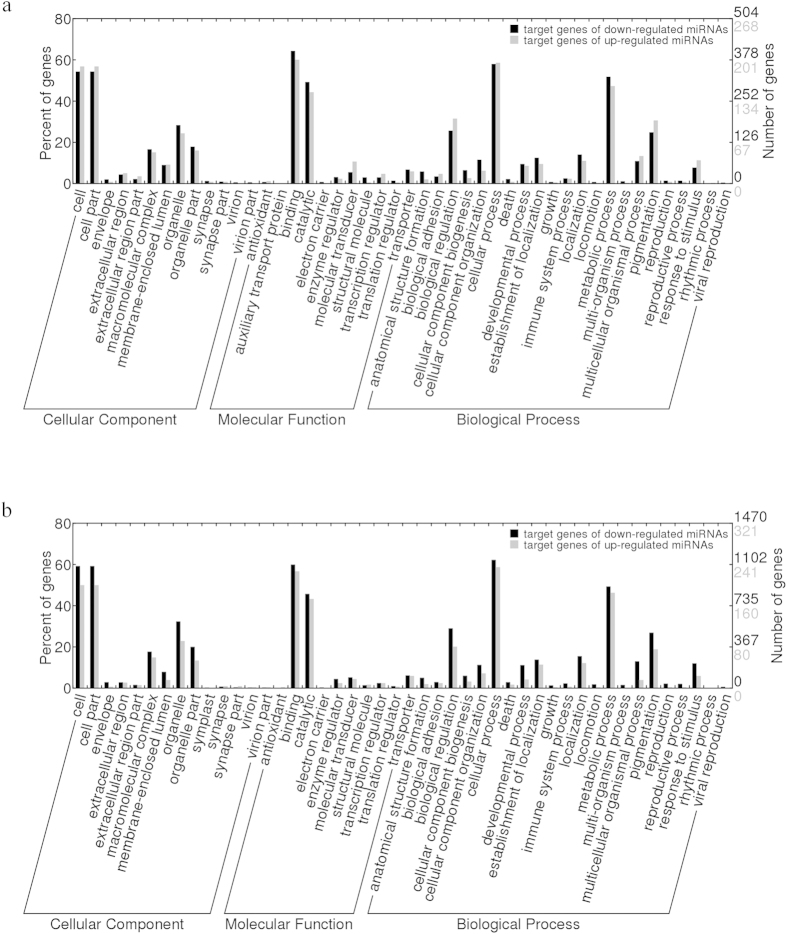
GO distribution of target genes of differentially expressed miRNAs of *C. gigas* (**a**) and *C. hongkongensis* (**b**). Red: target genes of up-regulated miRNAs; Purple: target genes of down-regulated miRNAs.

To better understand the functions of the identified miRNAs, candidate target genes were predicted using the genome of *C. gigas* and the transcriptome of *C. hongkongensis* as the reference transcript set by the miRanda software. In *C. gigas*, 472 target genes were predicted for the three up-regulated miRNAs and 880 target genes were identified for the three down-regulated miRNAs. In *C. hongkongensis*, 739 target genes were identified for the up-regulated miRNA and 3,607 target genes were identified for the down-regulated miRNAs. The GO assignment distribution of target genes was shown in Fig. 3. The biological functions of these target genes were further investigated using KEGG pathway analysis. More than 200 different pathways were found, and the most frequently represented pathways were involved in signal transduction and immune system (Fig. S1). This annotation of genes targeted by differentially expressed miRNAs suggests that the gene functions regulated by these miRNAs in response to how salinity stress are similar in the two species. The results indicate that there is no significant difference in gene function regulated by miRNAs in response to low salinity stress between the two oyster species.

The basic biological function of each putative target gene was classified based on the Gene Ontology enrichment and KEGG pathways analysis. The hypergeometric test was used to identify the overrepresented GO terms and KEGG pathways.

**Figure 4:**
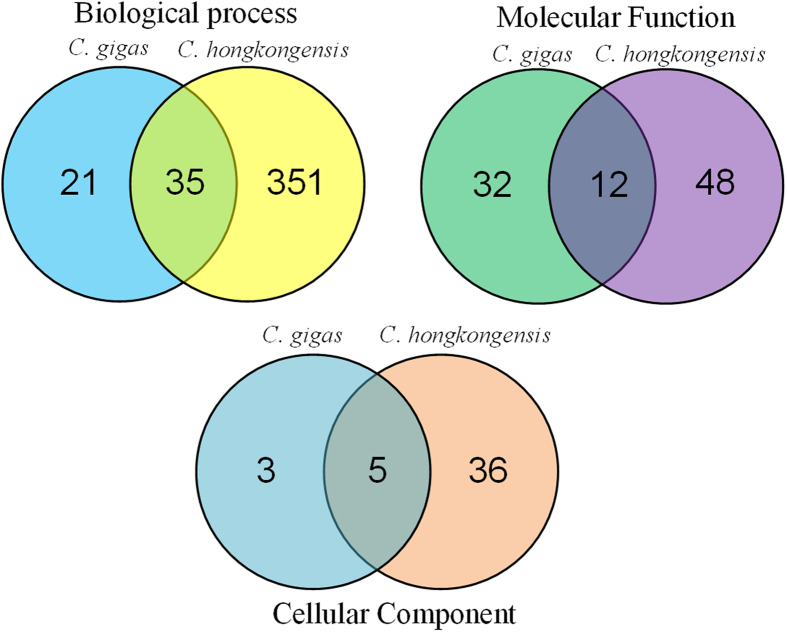
Venn diagram of shared enriched GO terms between *C. gigas* and *C. hongkongensis*.

The GO enrichment analysis provided several important biological processes enriched in both *C. gigas* and *C. hongkongensis*, such as microtubule-based process, cellular component movement, catabolic and metabolic process of purine nucleoside, and intracellular signal transduction. The enriched GO terms shared between the two species were shown in Fig. 4 and listed in Table S3. All the enriched GO terms were listed in Table S4 (for *C. gigas*) and Table S5 (for *C. hongkongensis*). Furthermore, the pathway analysis suggested that ECM-receptor interaction played an important regulatory role in both species. Several pathways were specifically enriched in the two species, such that cell adhesion molecules was enriched in the *C. gigas*, while MAPK signaling pathway and Focal adhesion were enriched in the *C. hongkongensis* (Table 3).

### Integrated expression analysis of microRNAs and their target mRNAs during osmotic stress

Gene expression regulation by miRNA relies on the miRNA-mRNA interactions. Based on the fact that miRNA regulate gene expression by inhibiting translation or inducing deadenylation of mRNA followed by their degradation^50^,^51^, expression profiling of miRNAs and mRNAs should reveal an inverse relationship if one regulates the other. Here we compared the predicted target genes of differentially expressed miRNAs in the two *Crassostrea* species by miRanda with differentially expressed mRNAs of the same samples^48^. As a result, expressions of two miRNAs, including chk-miR-2353 and chk-miR-3205, were negatively correlated with that of their corresponding target genes in response to low salinity stress (Table 4).

Of the osmoregulatory candidate genes identified in *C. gigas*^49^, 103 were identified in the target genes of differentially expressed miRNAs of *C. gigas* (Table S6) and 570 in those of *C. hongkongensis* based on 1237 transcripts of *C. hongkongensis* blasted to the osmoregulatory candidate genes of *C. gigas* (Table S7).

## Discussion

Since their roles in post-transcriptional regulation were unraveled, miRNAs have been extensively investigated in many organisms. Although little progress has been made in non-model species, the advances of high throughput sequencing technology provide unprecedented opportunities to efficiently characterize small RNA transcriptome in the molluscs. In this study, we conducted a high-throughput small RNA transcriptome sequencing in two oyster species in order to determine the roles that miRNA play in response to low salinity stress. For the consistent treatment to the two *Crassostrea* species, salinity 25‰ was considered as an optimal salinity level. The salinity level at their original habitat (the collection site) was 25‰ fluctuating with tide frequently. In previous studies, salinity 25‰ was used as the salinity of acclimation and proved as an appropriate salinity for physiological activities in both oyster species^45^,^52^,^53^,^54^. Salinity 8‰ is almost a extreme salinity for *C. gigas*^41^,^55^, which was used as treated salinity level for osmotic stress.

The small RNA transcriptome sequencing resulted in identification of 202 and 87 miRNAs from *C. gigas* and *C. hongkongensis*, respectively. There is a significant difference in the number of miRNAs identified in the two oyster species. It may be mainly due to the significant difference in abundance of the genomic information of the two oyster species. Further expression analysis indicated that six miRNAs in *C. gigas* and two miRNAs in *C. hongkongensis* were potentially involved in regulating the acclimation to low salinity stress conditions.

High-throughput sequencing provided sufficient and reliable genomic data for downstream small RNA analysis. The length distribution of reads (Fig. 1) in the four libraries was consistent with the observation in other aquatic organisms^20^,^25^,^56^, suggesting the conservation of miRNAs and the high quality data obtained for downstream analysis in this work. Using the genome of the Pacific oyster and the transcriptome of the Hongkong oyster as references for miRNA mapping, 202 miRNAs were identified in the gill of the *C. gigas*, with 137 being known miRNAs and 65 being novel miRNAs. Of the 137 known miRNAs, 71 known miRNAs were reported in a previous study that were identified from the haemocytes of *C. gigas*^25^. The remaining 66 known miRNAs could be a reflection of different miRNAs expression between the gill and haemocyte of 65 novel miRNAs, 16 miRNAs were also reported in haemocytes of the Pacific oyster^25^, and the remaining 49 novel miRNAs were discovered only in this study.

The expression of miRNAs under normal conditions was consistent with observations in other organisms. The miR-10 family (miR-10a, miR-10b, miR-10-5p), miR-184 family (miR-184, miR-184-3p), and miR-981 were the most abundant ones in the control groups of the two oyster species. Enhanced expression of miR-10a and miR-184 was also observed higher in haemocytes of *C. gigas*^25^, sea cucumber (*Apostichopus japonicas*)^20^,^21^ and Chinese mitten crab^57^. These miRNAs were conserved and had a long evolution history from Nephrozoa^58^, suggesting that they might play crucial roles in essential biological processes in various organisms^59^,^60^,^61^.

Cgi-miR-183, cgi-miR-184-3p, cgi-miR-2353, cgi-miR-1984, cgi-miR-92 and scaffold43364_10952 were differentially expressed in the gills of *C. gigas* under osmotic stress, while chk-miR-3205 and chk-miR-2353 were differentially expressed in the gills of *C. hongkongensis*. Previous studies reported that miR-183 was involved in response to environmental stress. The miR-183 functioned as a regulator of target genes to activate the c-Jun N-terminal kinase mitogen-activated protein kinase pathway and regulate the pathways of apoptosis^62^,^63^. Moreover, members of miR-183 family were found to be expressed in innervated regions of invertebrate deuterostomes^64^ and regulate down-stream effectors functioning in actin cytoskeleton and plasma membrane^65^. It is reported that cytoskeleton rearrangement may maintain internal and external osmotic pressure balance when the Pacific oyster responds quickly to osmotic stress^34^. In this study, cgi-miR-183 was significantly down-regulated under low osmotic conditions compared to normal conditions, which may regulate the genes involved in cytoskeleton to response to salinity stress. The miR-184-3p is a specific miRNA that can modify the microRNP (microribonucleoproteins) function and relieve the repression induced by stress^66^. The miR-92, belonging to miR-17-19 cluster, is an immune-related miRNA^67^, and is associated with pro-proliferative and anti-apoptotic properties^68^. Notably, the differentially expressed miRNAs with known functions identified in the present study are mainly involved in immune-related function and response to abiotic stress. However, the function of cgi-miR-1984, miR-2353, chk-miR-3205 and scaffold43364_10952 are still unknown. The miR-1984 was first discovered as a gastropod-specific miRNA gene^58^ and was only found in *Lottia gigantea* and *Haliotis rufescens*. However, the identification of miR-1984 in the haemocytes of *C. gigas* in the previous study^25^ and gills of *C. gigas* in this study suggested that miR-1984 was not gastropod-specific but could be mollusk-specific. The expression of miR-1984 increased significantly in response to low salinity stress, heat stress and bacteria challenge suggesting that it could be implicated in certain physiological functions such as oxidation reduction and energy metabolism. The miR-2353 and miR-3205 were previously discovered in cattle^69^ and silkworm^70^. The target genes of the three miRNAs (miR-1984, miR-2353, and miR-3205) might be implicated in signal transduction and proteolysis for energy metabolism and other physiological functions. Further functional analysis are needed to elucidate their roles in molluscs.

Integrated expression analysis of miRNAs and their target genes identified the interaction of miRNAs and mRNAs involved in regulating specific biological processes. The enrichment analysis of putative target genes revealed the relationship between miRNAs expression and response to low-osmotic stress in the two oyster species. Based on the results of differentially expressed transcripts in *C. gigas* and *C. hongkongensis*^48^, we were able to identity a total of five functional miRNA-mRNA interaction pairs in response to low salinity stress (Table 4). As anticipated, all identified miRNA-mRNA interactions showed that the expression of miRNAs had negatively correlationships with that of their mRNA targets, consistent with the observations that miRNAs regulate target gene expression by repressing their targets^71^ through transcript cleavage or translation repression.

The chk-miRNA-2353 appeared to target *ATP grasp domain containing protein 1* (ATPGD1) and *cAMP responsive element binding protein like 2* (CREBL2). ATPGD1 is involved in metabolic process and catalyzes the degradation of beta-alanine in KEGG to maintain osmotic equilibrium under hypo-osmotic stress in the oysters. Its expression was found to increase significantly in the *C. gigas* on the 7^th^ day after hypo-osmotic stress, and reached the highest level under the condition with salinity of 10^55^. The CREB family was reported to be involved in immune processes in mollusca^72^,^73^. The chk-miRNA-3205 targets *hygromycin phosphotransferase*, *Replication factor A protein 1* (RFP1) and *von Willebrand factor D and EGF domain containing protein* (VWDE). RFP1 has zinc finger domain, which has been reported to be involved in stress response and apoptosis in previous studies^34^,^74^,^75^. VWDE was putatively related to immunity and stress^76^,^77^. These miRNA-mRNA interaction pairs deserve future investigation.

Dramatic differences in expression profiles of miRNAs were observed between the two oyster species. We detected six miRNAs and 48 corresponding target genes in the *C. gigas*, while two miRNAs and 408 corresponding target genes were identified in the *C. hongkongensis*, in response to hypo-osmotic stress. Five miRNA-mRNA interaction pairs were found in *C. hongkongensis*, while none was identified in *C. gigas*. This result was far below observation reported in previous studies^75^,^78^. Possible reasons to explain this observation include: (1) the computationally-predicted targets do not represent the actually existing interactions, (2) strict filtering criterion may exclude certain actual interaction pairs, (3) the expression of specific genes are regulated at temporal and spatial levels, and most targets may not be regulated at this point, and (4) there is less interaction pairs in the *C. gigas* than that in the *C. hongkongensis*, which is consistent with the observation that *C. hongkongensis* had higher levels of tolerance than that of *C. gigas* in response to acute hypo-osmotic stress. Similarly, comparison between the control group of *C. gigas* and *C. hongkongensis* also showed a dramatic difference, indicating the different tolerance to low salinity stress between the two oyster species. Moreover, miR-2353 may be the main miRNAs to resist low osmotic stress, which was the only one miRNA down-regulated in both species of the osmoregulatory candidate genes identified in *C. gigas*^49^, the targets of differentially expressed miRNAs in *C. gigas* and *C. hongkongensis* that were identified in osmoregulatory candidates may be the candidate genes regulated by miRNAs in response to osmotic stress. There were more osmoregulatory candidates regulated by miRNA in *C. hongkongensis* than in *C. gigas*. The results indicated that *C. hongkongensis* can regulate more osmoregulatory genes in response to acute hypo-osmotic stress.

## Materials and Methods

### Ethics statement

The *C. hongkongensis* and *C. gigas* are not endangered or protected species, and there is no requirement for permission to perform experiments involving the two oysters (invertebrate) in China.

### Sample collection

The samples used in this work were same as described in our previous RNA-seq study^48^. In brief, adult individuals of *C. gigas* (2 year-old) were collected from Weihai (Shandong, China), and adult *C. hongkongensis* (2 year-old) were collected from Zhanjiang (Guangdong, China) in 2010. One hundred oysters of each species were acclimated in an aquarium tank supplied with sand-filtered seawater at ambient temperature (18 ± 1 °C) and salinity (25‰). After acclimation for a week, the two species of oysters were divided into two groups (control group and treatment group), respectively. Control groups were kept in filtered seawater with optimal salinity 25‰, while treatment groups were exposed to low osmotic filtered seawater with salinity of 8‰. In order to ensure the free exchange of seawater between the inside and outside of the shell, a part of the shell edge (about 10 mm long and 5 mm wide) of each specimen was chipped away. After 8 hours, six oysters from each group were randomly selected, and the gills were dissected for RNA extraction.

### Small RNA library preparation and sequencing

Experimental protocols for the cDNA normalization sequences were performed according to the manufacturer’s technical instructions. Firstly, the total RNA was isolated from gill tissues with TRIzol reagent (Invitrogen), and the RNA of 15–40 nt was purified by the 15% Polyacrylamide gel. Equal amounts of the high-quality small RNA samples from six individuals of each group were then pooled for cDNA library preparation using the TruSeq Small RNA sample Preparation Kit (Illumina), respectively. Then these small RNAs were ligated sequentially to 5′ and 3′ adapters and used SuperScript II Reverse Transcriptase (Life Technologies) to synthesize the first-strand cDNA. The double-strand cDNA was performed followed by PCR amplification.

The purified PCR products were used for cluster generation by TruSeq PE Cluster Kit (Illumina) and then was sequenced on Illumina Hiseq 2000 following the manufacturer’s instructions. Raw reads obtained from Illumina sequencing have been deposited in the National Center for Biotechnology Information Short Reads Archive ( http://www.ncbi.nlm.nih.gov/srawebsite) under accession number SRP049540.

### Data processing

The raw reads were subject to the program FastQC^79^ ( http://www.bioinformatics.babraham.ac.uk/projects/fastqc/) to assess the quality of sequencing data and trim low quality reads and adaptor sequence. The reads with high sequencing quality and ranging from 15–30nt in length were annotated by searching against the GenBank database^80^ and the Rfam database^81^. In addition, the clean reads of *C. gigas* were aligned to the Pacific oyster genome (GenBank accession: AFTI00000000.1, http://metazoa.ensembl.org/Crassostrea_gigas/Info/Index) while the reads of *C. hongkongensis* were aligned to the transcriptome of the Hongkong oyster^48^ using bowtie software^82^ with no more than 2 nt mismatches to filter the reads.

### Analysis of conserved and novel miRNAs

Reads mapped to either non-miRNA in Rfam (such as rRNAs, tRNAs, snoRNAs etc.) or oyster mRNAs, were excluded for further analysis. The remaining reads were aligned against the miRBase 20.0^83^ and the oyster genome for conserved and novel miRNA identification combined with stem-loop structure prediction. The clean reads were mapped to mature miRNA and hairpin sequences in miRBase 20.0 with complete matches to identify conserved miRNAs. Reads that did not match miRBase database were marked as unannotated. The unannotated data sets were aligned with the oyster genomic sequences to predict novel miRNAs, using miRDeep2 software^84^ with the prediction of the secondary structure.

### Differential expression analysis of miRNAs

To compare miRNAs expression data between the control and treatment groups, read counts for each identified miRNA were normalized to the total number of reads in each given sample. Two methods, DESeq^85^ and edgeR^86^, were used to determine the differential expression among the experimental groups. These two methods are all have type-I error control^87^. EdgeR detected differentially expressed transcripts at lower sensitivity to DESeq, while DESeq returned more false-positive transcripts^88^. A common practice to boost the result accuracy is to use more than one method and then to combine their results. So only differentially expressed miRNAs identified by both methods were considered for further analysis. The fold change of miRNAs was calculated as the ratio of read counts in the treatment group to the read counts in the control group followed by transformation of log_2._ The miRNAs with the absolute value of log_2_ (fold change) ≥1.0, and q values <0.05 were considered as significantly differentially expressed.

### Target gene prediction and functional annotation

The 3′UTR sequences of oyster protein-coding genes were retrieved based on the oyster genomic sequences and annotation information. The target genes of oyster miRNAs were predicted using the miRanda algorithm (score ≥160, free energy ≤−25 kcal/mol).

The target sequences were annotated using Blast2GO software^89^ for assigning GO terms to investigate their putative functions. The GO terms were plotted through Web Gene Ontology Annotation Plot (WEGO)^90^. Moreover, the target genes were also annotated using Kyoto Encyclopedia of Genes and Genomes^91^. To determine the possible overlapping of biological functions among these miRNAs significantly overrepresented GO terms and KEGG pathways were searched using the GOstat^92^ package and the GSEABase package^93^ with a P-value cutoff of 0.01.

Based on the results of miRanda algorithm, the differentially expressed mRNA from transcriptome data^48^ were compared with the target genes of differentially expressed miRNAs. The genes that were found to be differentially expressed in *C. gigas* adults in response to six different salinity treatments when compared to a control salinity of 30 Table S21 in Zhang *et al.*^49^ were considered as osmoregulatory candidates^35^. Additionally, we quantified the number of target genes of differentially expressed miRNAs that were included in osmoregulatory candidate genes.

### qRT-PCR validation of miRNA expression

The miRNA were extracted and purified from gill tissues using High Pure miRNA Isolation Kit (Roche) according to the manufacturer’s instructions. The reverse transcription was carried out using on miScript II RT Kit (QIAGEN). The synthesis reaction was incubated for 60 min at 37 °C, and terminated by heating at 95 °C for 5 min to inactivate enzyme reaction.

The qRT-PCR was performed using the miScript SYBR Green PCR Kit (QIAGEN). The reactions were carried out in a total volume of 25 μl containing 2.5 μl of diluted cDNA, 2.5 μl of each primer, and 12.5 μl SYBR Green PCR Master Mix with the following cycling profile: 95 °C for 15 min for polymerase activation, followed by 45 cycles at 94 °C for 15s, 55 °C for 30 s, 70 °C for 30 s. Eight miRNA fragments were amplified using specific forward primers (Table 5) and universal reverse primers and 5S fragment were used as an internal control. Each sample was processed in triplicate and conducted with Roche Lightcycler 480 (Roche). All data was analyzed using 2 ^–ΔΔCt^ method.

## Conclusions

This is the first report to investigate the expression profiles of small RNAs in response to acute hypo-osmotic stress in oysters. A total of 202 and 87 miRNAs were identified from *C. gigas* and *C. hongkongensis*, respectively. Differential expression analysis of the miRNAs suggested that miRNAs played conspicuous roles in response to low salinity stress. Based on the GO annotation of target genes, miRNA mainly participate in the biological processes including microtubule-based process and cellular component movement, etc. Meanwhile the different expression patterns of miRNAs and the miRNA-mRNA interaction pairs indicated the differences in adaptation to hypo-osmotic stress between two oyster species. Further studies are required to understand the biological functions of miRNAs, and focus on the responsible miRNAs and Mollusca-specific miRNAs in response to osmotic stress. Additionally, increasing experimental sets would generate more conclusions.

## Additional Information

**How to cite this article**: Zhao, X. *et al.* High throughput sequencing of small RNAs transcriptomes in two *Crassostrea* oysters identifies microRNAs involved in osmotic stress response. *Sci. Rep.* **6**, 22687; doi: 10.1038/srep22687 (2016).

## Supplementary Material

Supplementary Information

Supplementary Table S1

Supplementary Table S2

Supplementary Table S3

Supplementary Table S4

Supplementary Table S5

Supplementary Table S6

Supplementary Table S7

## Figures and Tables

**Table 1:** Summary of small RNA transcriptome sequencing of gill tissues in *C. gigas* and *C. hongkongensis.*

Category	C. gigas				C. hongkongensis			
	Control		Treated		Control		Treated	
	Unique	Redundant	Unique	Redundant	Unique	Redundant	Unique	Redundant
miRNA	1,878	9,150,470 (71.38%)	2,108	9,492,885 (67.12%)	1,223	14,682,335 (68.04%)	1,401	13,579,598 (65.01%)
rRNA*	5,968	86,221 (0.67%)	8,297	146,244 (1.03%)	7,552	143,791 (0.67%)	9,732	213,415 (1.02%)
tRNA*	976	31,809 (0.25%)	1,491	88,173 (0.62%)	136	1,978 (0.009%)	220	3,367 (002%)
Other Rfam category*	1,888	6,843 (0.05%)	2,472	12,042 (0.09%)	2,396	9,891 (0.04%)	2,832	15,273 (0.07%)
Unannotated	56,073	823,478 (6.42%)	68,386	1,081,813 (7.65%)	103,777	892,043 (4.13%)	121,226	1,169,750 (5.60%)
Mapping to genome*	66,783	10,098,821 (78.78%)	82,754	10,821,155 (76.51%)	115,084	15,730,038 (72.89%)	135,411	14,981,403 (71.72%)
Clean reads	320,436	12,819,335	429,972	14,142,838	464,695	21,580,148	572,293	20,889,308
Raw data	13,268,157		14,682,519		22,288,517		21,645,549	

^*^excluded for further analysis.

**Table 2:** Differentially expressed miRNAs identified from the *C. gigas* and *C. hongkongensis* between control and treatment groups.

miRNA	Read counts (Control group)	Normalized read counts (Control group)	Reads_counts (Treatment group)	Normalized read counts (Treatment group)	Log2(foldchange)	P-value	Species
scaffold43364_10952	23	23.62598138	588	572.420666	4.598629596	3.74E-05	*C. gigas*
cgi-miR-2353	72	73.6333131	2	1.955636572	−5.234648421	0.00174025	*C. gigas*
cgi-miR-1984	164,760	169,244.204	534,981	520,806.4292	1.62164083	0.00184559	*C. gigas*
cgi-miR-183	2,160	2,208.999393	908	887.8590035	−1.31499053	0.02035668	*C. gigas*
cgi-miR-92-3p	73,928	75,605.0496	165,791	162,113.4714	1.100449483	0.02711921	*C. gigas*
cgi-miR-184-3p	601,642	618,016.6388	308,210	300,043.8325	−1.042472405	0.04226714	*C. gigas*
chk-miR-3205	2	1.992031841	110	110.4400018	5.792878298	8.05E-09	*C. hongkongensis*
chk-miR-2353	118	117.5298786	4	4.016000064	−4.871124465	3.85E-08	*C. hongkongensis*

**Table 3:** List of significantly enriched KEGG pathways of candidate targets of differentially expressed miRNAs in *C. gigas* and *C. hongkongensis*.

KEGG pathway	Term	Gene Counts	Gene number of Pathway	P-value	species
Map04514	Cell adhesion molecules (CAMs)	6	12	0.000102	*C. gigas*
Map04512	ECM-receptor interaction	7	27	0.002859	*C. gigas*
Map04010	MAPK signaling pathway	30	113	0.000105	*C. hongkongensis*
Map04510	Focal adhesion	24	85	0.000185	*C. hongkongensis*
Map04515	Rap1 signaling pathway	20	79	0.002729	*C. hongkongensis*
Map04512	ECM-receptor interaction	8	21	0.003862	*C. hongkongensis*
Map00410	Beta-Alanine metabolism	9	27	0.006183	*C. hongkongensis*
Map05164	Influenza A	17	69	0.007415	*C. hongkongensis*
Map04062	Chemokine signaling pathway	14	54	0.009128	*C. hongkongensis*

**Table 4:** Interaction pairs of differentially expressed miRNAs and predicted mRNAs in *C. hongkongensis*.

miRNA name	Target Gene-Interaction	miRNA expression level		mRNA expression level		Target annotation
		log2(fold change)	P-value	log_2_(fold change)	P-value	
chk-miRNA-2353	comp106174	−4.87	3.85E-08	5.75	9.30E-06	ATP grasp domain containing protein 1
	comp24781	−4.87	3.85E-08	6.05	3.15E-05	cAMP responsive element binding protein like 2
chk-miRNA-3205	comp41123	5.79	8.05E-09	HC	6.90E-04	Hygromycin phosphotransferase
	comp54488	5.79	8.05E-09	HC	2.38E-05	Replication factor A protein 1
	comp62265	5.79	8.05E-09	−6.40	1.44E-05	von Willebrand factor D and EGF domain containing protein

HC: mRNA expressed only in HC group with no value of log_2_ (fold change).

**Table 5:** The primer sequences used for qRT-PCR in this study.

Primer	Sequence(5′-3′)
5S	TTGGATGGGTGACCGCCTG
miR-2353 (*C.gigas*)	TCTGTACTGCAGAATATCCAATATC
miR-1984	TGCCCTATCCGTCAGTCG
miR-92	GCCGGGACGAGTGCAATA
scaffold43364_10952	GTGCTCATTTGTCGAAACTGT
miR-183	TGGGAATGGCACTGGTAGAATTC
miR-184-3p	CCTTATCAGTTCTCCGTCCA
miR-3205	CACTCGGTGGACTGCTCG
miR-2353 (*C.hongkongensis*)	ACACTTCTCCAGTACAGAGGATCT

## References

[b1] Obernosterer G., Leuschner P. J. F., Alenius M. & Martinez J. Post-transcriptional regulation of microRNA expression. RNA 12, 1161–1167 (2006). 16738409 10.1261/rna.2322506 PMC1484437

[b2] Wienholds E. *et al.* MicroRNA expression in zebrafish embryonic development. Science 309, 310–311 (2005). 15919954 10.1126/science.1114519

[b3] Wienholds E. & Plasterk R. H. A. MicroRNA function in animal development. FEBS Lett. 579, 5911–5922 (2005). 16111679 10.1016/j.febslet.2005.07.070

[b4] Li M. *et al.* High throughput sequencing of two celery varieties small RNAs identifies microRNAs involved in temperature stress response. BMC Genomics 15, 242 (2014). 24673837 10.1186/1471-2164-15-242 PMC3986682

[b5] Yan B., Zhao L. H., Guo J.-T. & Zhao J. L. miR-429 regulation of osmotic stress transcription factor 1 (OSTF1) in tilapia during osmotic stress. Biochem Bioph Res Co 426, 294–298 (2012). 10.1016/j.bbrc.2012.08.029 22940129

[b6] Gracias D. T. & Katsikis P. D. In Crossroads Between Innate and Adaptive Immunity III Vol. 780 Advances in Experimental Medicine and Biology (eds Pulendran B., Katsikis P. D. & Schoenberger S. P. ) 15–26 (2011).

[b7] Martín-Gómez L., Villalba A., Kerkhoven R. H. & Abollo E. Role of microRNAs in the immunity process of the flat oyster *Ostrea edulis* against bonamiosis. Infect Genet. Evol. 27, 40–50 (2014). 25008434 10.1016/j.meegid.2014.06.026

[b8] Zhang B., Wang Q. & Pan X. MicroRNAs and their regulatory roles in animals and plants. J. Cell Physiol. 210, 279–289 (2007). 17096367 10.1002/jcp.20869

[b9] Friedman R. C., Farh K. K.-H., Burge C. B. & Bartel D. P. Most mammalian mRNAs are conserved targets of microRNAs. Genome Res. 19, 92–105 (2009). 18955434 10.1101/gr.082701.108 PMC2612969

[b10] Bellin D. *et al.* Combining next-generation pyrosequencing with microarray for large scale expression analysis in non-model species. BMC Genomics 10, 555 (2009). 19930683 10.1186/1471-2164-10-555 PMC2790472

[b11] Ekblom R. & Galindo J. Applications of next generation sequencing in molecular ecology of non-model organisms. Heredity 107, 1–15 (2010). 21139633 10.1038/hdy.2010.152 PMC3186121

[b12] Lu J. *et al.* The birth and death of microRNA genes in *Drosophila*. Nat. Genet. 40, 351–355 (2008). 18278047 10.1038/ng.73

[b13] Leong I. U. S., Lan C.-C., Skinner J. R., Shelling A. N. & Love D. R. *In vivo* testing of microRNA-mediated gene knockdown in zebrafish. J. Biomed. Biotechnol. 350352 (2012). 22500088 10.1155/2012/350352 PMC3303736

[b14] Song Q. X. *et al.* Identification of miRNAs and their target genes in developing soybean seeds by deep sequencing. BMC Plant Biol. 11, 5 (2011). 21219599 10.1186/1471-2229-11-5 PMC3023735

[b15] Qi P., Guo B., Zhu A., Wu C. & Liu C. Identification and comparative analysis of the *Pseudosciaena crocea* microRNA transcriptome response to poly (I: C) infection using a deep sequencing approach. Fish Shellfish Immun. 39, 483–491 (2014). 10.1016/j.fsi.2014.06.009 24945573

[b16] Yan B., Wang Z., Zhu C., Guo J. & Zhao J. MicroRNA repertoire for functional genome research in tilapia identified by deep sequencing. Mol. Biol. Rep. 41, 4953–4963 (2014). 24752404 10.1007/s11033-014-3361-9

[b17] Bizuayehu T. T., Johansen S. D., Puvanendran V., Toften H. & Babiak I. Temperature during early development has long-term effects on microRNA expression in Atlantic cod. BMC Genomics 16, 305 (2015). 25881242 10.1186/s12864-015-1503-7 PMC4403832

[b18] Gong G. *et al.* Expression Profiling analysis of the microrna response of *Cynoglossus semilaevis* to vibrio anguillarum and other stimuli. Marine Biotechnol. 17, 338–352 (2015). 10.1007/s10126-015-9623-2 25715708

[b19] Ma H. *et al.* MicroRNA expression profiles from eggs of different qualities associated with post-ovulatory ageing in rainbow trout (*Oncorhynchus mykiss*). BMC Genomics 16, 201 (2015). 25885637 10.1186/s12864-015-1400-0 PMC4374207

[b20] Li C. *et al.* Characterization of skin ulceration syndrome associated microRNAs in sea cucumber *Apostichopus japonicus* by deep sequencing. Fish Shellfish Immun. 33, 436–441 (2012). 10.1016/j.fsi.2012.04.013 22626809

[b21] Chen M., Zhang X., Liu J. & Storey K. B. High-throughput sequencing reveals differential expression of miRNAs in intestine from sea cucumber during aestivation. PloS One 8, e76120 (2013). 24143179 10.1371/journal.pone.0076120 PMC3797095

[b22] Zhang P. *et al.* De novo assembly of the sea cucumber *Apostichopus japonicus* hemocytes transcriptome to identify miRNA targets associated with skin ulceration syndrome. PloS One 8, e73506 (2013). 24069201 10.1371/journal.pone.0073506 PMC3772007

[b23] Mi X., Wei Z., Zhou Z. & Liu X. Identification and profiling of sex-biased microRNAs from sea urchin *Strongylocentrotus nudus* gonad by Solexa deep sequencing. Comp. Biochem. Phy. D 10, 1–8 (2014). 10.1016/j.cbd.2014.01.001 24486540

[b24] Wang H. *et al.* Characterization and expression analysis of micrornas in the tube foot of sea cucumber *Apostichopus japonicus*. PloS One 9, e111820 (2014). 25372871 10.1371/journal.pone.0111820 PMC4221132

[b25] Zhou Z. *et al.* The identification and characteristics of immune-related microRNAs in haemocytes of oyster *Crassostrea gigas*. PloS One 9, e88397 (2014). 24516648 10.1371/journal.pone.0088397 PMC3916443

[b26] Jiao Y. *et al.* Identification and characterization of microRNAs in Pearl Oyster *Pinctada martensii* by solexa deep sequencing. *Marine* Biotechnol 16, 54–62 (2014). 10.1007/s10126-013-9528-x 23877619

[b27] Bao Y., Zhang L., Dong Y. & Lin Z. Identification and comparative analysis of the *Tegillarca granosa* haemocytes microRNA transcriptome in response to Cd using a deep sequencing approach. PloS One 9, e93619 (2014). 24690903 10.1371/journal.pone.0093619 PMC3972184

[b28] Xu F. *et al.* Identification of conserved and novel micrornas in the pacific oyster *Crassostrea gigas* by deep sequencing. PloS One 9, e104371 (2014). 25137038 10.1371/journal.pone.0104371 PMC4138081

[b29] Chen H. *et al.* The comprehensive immunomodulation of NeurimmiRs in haemocytes of oyster *Crassostrea gigas* after acetylcholine and norepinephrine stimulation. BMC Genomics 16, 942 (2015). 26576764 10.1186/s12864-015-2150-8 PMC4650145

[b30] Leung A. K. L. & Sharp P. A. MicroRNA functions in stress responses. Mol. Cell 40, 205–215 (2010). 20965416 10.1016/j.molcel.2010.09.027 PMC2996264

[b31] Chapman R. W. *et al.* The transcriptomic responses of the eastern oyster, *Crassostrea virginica*, to environmental conditions. Mol. Ecol. 20, 1431–1449 (2011). 21426432 10.1111/j.1365-294X.2011.05018.x

[b32] Chaney M. L. & Gracey A. Y. Mass mortality in Pacific oysters is associated with a specific gene expression signature. Mol. Ecol. 20, 2942–2954 (2011). 21672066 10.1111/j.1365-294X.2011.05152.x

[b33] Martin-Gomez L., Villalba A. & Abollo E. Identification and expression of immune genes in the flat oyster *Ostrea edulis* in response to bonamiosis. Gene 492, 81–93 (2012). 22085815 10.1016/j.gene.2011.11.001

[b34] Zhao X., Yu H., Kong L. & Li Q. Transcriptomic responses to salinity stress in the pacific oyster *Crassostrea gigas*. PloS One 7, e46244 (2012). 23029449 10.1371/journal.pone.0046244 PMC3459877

[b35] Eierman L. E. & Hare M. P. Transcriptomic analysis of candidate osmoregulatory genes in the eastern oyster *Crassostrea virginica*. BMC Genomics 15, 503 (2014). 24950855 10.1186/1471-2164-15-503 PMC4101419

[b36] Eierman L. E. & Hare M. P. Reef-specific patterns of gene expression plasticity in Eastern oysters (*Crassostrea virginica*). J. Hered. 00, esv057 (2015). 10.1093/jhered/esv057 26245921

[b37] Tomanek L., Zuzow M. J., Ivanina A. V., Beniash E. & Sokolova I. M. Proteomic response to elevated *P*_*CO2*_ level in eastern oysters, *Crassostrea virginica*: evidence for oxidative stress. J. Exp. Biol. 214, 1836–1844 (2011). 21562170 10.1242/jeb.055475

[b38] Dineshram R. *et al.* Analysis of Pacific oyster larval proteome and its response to high-CO2. Mar. Pollut. Bull. 64, 2160–2167 (2012). 22921897 10.1016/j.marpolbul.2012.07.043

[b39] Muralidharan S., Thompson E., Raftos D., Birch G. & Haynes P. A. Quantitative proteomics of heavy metal stress responses in Sydney rock oysters. Proteomics 12, 906–921 (2012). 22539440 10.1002/pmic.201100417

[b40] Corporeau C., Tamayo D., Pernet F., Quéré C. & Madec S. Proteomic signatures of the oyster metabolic response to herpesvirus OsHV-1 μVar infection. J. Proteomics 109, 176–187 (2014). 25009143 10.1016/j.jprot.2014.06.030

[b41] Zhang Y. *et al.* Proteomic basis of stress responses in the gills of the Pacific oyster *Crassostrea gigas*. J. Proteome Res. 14, 304–317 (2014). 25389644 10.1021/pr500940s

[b42] Guo X., He Y., Zhang L., Lelong C. & Jouaux A. Immune and stress responses in oysters with insights on adaptation. Fish Shellfish Immun. 46, 107–119 (2015). 10.1016/j.fsi.2015.05.018 25989624

[b43] Wang H. & Guo X. Identification of *Crassostrea ariakensis* and related oysters by multiplex species-specific PCR. J. Shellfish Res. 27, 481–487 (2008).

[b44] Pauley G. B., Van Der Raay B. & Troutt D. Species Profiles: Life Histories and Environmental Requirements of Coastal Fishes and Invertebrates (Pacific Northwest), Pacific Oyster. In: Fish and Wildlife Service Biological Report (1988)

[b45] Zhang Z. & Zhang Q. Molecular cloning, characterization and expression of heat shock protein 70 gene from the oyster *Crassostrea hongkongensis* responding to thermal stress and exposure of Cu^2+^and malachite green. Gene 497, 172–180 (2012). 22310388 10.1016/j.gene.2012.01.058

[b46] Liu F., Rainbow P. S. & Wang W. Inter-site differences of zinc susceptibility of the oyster *Crassostrea hongkongensis*. Aquat. Toxicol. 132, 26–33 (2013). 23454307 10.1016/j.aquatox.2013.01.022

[b47] Hosoi M. *et al.* Taurine transporter from the giant Pacific oyster *Crassostrea gigas*: function and expression in response to hyper-and hypo-osmotic stress. Fish Sci. 73, 385–394 (2007).

[b48] Zhao X., Yu H., Kong L., Liu S. & Li Q. Comparative transcriptome analysis of two oysters, *Crassostrea gigas* and *Crassostrea hongkongensis* provides insights into adaptation to hypo-osmotic conditions. PloS One 9, e111915 (2014). 25369077 10.1371/journal.pone.0111915 PMC4219811

[b49] Zhang G. *et al.* The oyster genome reveals stress adaptation and complexity of shell formation. Nature 490, 49–54 (2012). 22992520 10.1038/nature11413

[b50] Bartel D. P. MicroRNAs: target recognition and regulatory functions. Cell 136, 215–233 (2009). 19167326 10.1016/j.cell.2009.01.002 PMC3794896

[b51] Sayed D. & Abdellatif M. MicroRNAs in development and disease. Physiol. Rev. 91, 827–887 (2011). 21742789 10.1152/physrev.00006.2010

[b52] Quayle D. B. Pacific oyster culture in British Columbia. Department of Fisheries and Oceans (1988).

[b53] Gagnaire B., Frouin H., Moreau K., Thomas-Guyon H. & Renault T. Effects of temperature and salinity on haemocyte activities of the Pacific oyster, *Crassostrea gigas* (Thunberg). Fish Shellfish Immun. 20, 536–547 (2006). 10.1016/j.fsi.2005.07.003 16182565

[b54] Zhang Y. *et al.* Artificial interspecific backcrosses between the hybrid of female *Crassostrea hongkongensis* × male *C. gigas* and the two parental species. Aquaculture 450, 95–101 (2016).

[b55] Meng J. *et al.* Genome and transcriptome analyses provide insight into the euryhaline adaptation mechanism of *Crassostrea gigas*. PloS One 8, e58563 (2013). 23554902 10.1371/journal.pone.0058563 PMC3595286

[b56] Kitano J., Yoshida K. & Suzuki Y. RNA sequencing reveals small RNAs differentially expressed between incipient Japanese threespine sticklebacks. BMC Genomics 14, 214 (2013). 23547919 10.1186/1471-2164-14-214 PMC3637797

[b57] Song Y., Shi L., Liu Z. & Qiu G. Global analysis of the ovarian microRNA transcriptome: implication for miR-2 and miR-133 regulation of oocyte meiosis in the Chinese mitten crab, *Eriocheir sinensis* (Crustacea: Decapoda). BMC Genomics 15, 547 (2014). 24984770 10.1186/1471-2164-15-547 PMC4092226

[b58] Wheeler B. M. *et al.* The deep evolution of metazoan microRNAs. Evol. Dev. 11, 50–68 (2009). 19196333 10.1111/j.1525-142X.2008.00302.x

[b59] Lund A. H. miR-10 in development and cancer. Cell Death Differ. 17, 209–214 (2009). 19461655 10.1038/cdd.2009.58

[b60] Wu J., Bao J., Wang L., Hu Y. & Xu C. MicroRNA-184 downregulates nuclear receptor corepressor 2 in mouse spermatogenesis. BMC Dev. Biol. 11, 64 (2011). 22017809 10.1186/1471-213X-11-64 PMC3227627

[b61] Pushpavalli S. N. *et al.* Argonaute-1 functions as a mitotic regulator by controlling Cyclin B during *Drosophila* early embryogenesis. FASEB J. 28, 655–666 (2014). 24165481 10.1096/fj.13-231167 PMC6191000

[b62] Kovalchuk O. *et al.* microRNAome changes in bystander three-dimensional human tissue models suggest priming of apoptotic pathways. Carcinogenesis 31, 1882–1888 (2010). 20643754 10.1093/carcin/bgq119 PMC2950932

[b63] Patel M. *et al.* The miR-183/Taok1 target pair is implicated in cochlear responses to acoustic trauma. PloS One 8, e58471 (2013). 23472202 10.1371/journal.pone.0058471 PMC3589350

[b64] Pierce M. L. *et al.* MicroRNA-183 family conservation and ciliated neurosensory organ expression. Evol. Dev. 10, 106–113 (2008). 18184361 10.1111/j.1525-142X.2007.00217.x PMC2637451

[b65] Wang G., Mao W. & Zheng S. MicroRNA-183 regulates Ezrin expression in lung cancer cells. FEBS Lett. 582, 3663–3668 (2008). 18840437 10.1016/j.febslet.2008.09.051

[b66] Vasudevan S. Posttranscriptional upregulation by microRNAs. WIRS-RNA 3, 311–330 (2012). 10.1002/wrna.121 22072587

[b67] Ou J. *et al.* Identification and comparative analysis of the *Eriocheir sinensis* microRNA transcriptome response to *Spiroplasma eriocheiris* infection using a deep sequencing approach. Fish Shellfish Immun. 32, 345–352 (2012). 10.1016/j.fsi.2011.11.027 22166732

[b68] Pogribny I. P. *et al.* Induction of microRNAome deregulation in rat liver by long-term tamoxifen exposure. Mutat. Res. 619, 30–37 (2007). 17343880 10.1016/j.mrfmmm.2006.12.006

[b69] Glazov E. A. *et al.* Repertoire of bovine miRNA and miRNA-like small regulatory RNAs expressed upon viral infection. PloS One 4, e6349 (2009). 19633723 10.1371/journal.pone.0006349 PMC2713767

[b70] Cai Y. *et al.* Novel microRNAs in silkworm (*Bombyx mori*). Funct. Integr. Genomics 10, 405–415 (2010). 20229201 10.1007/s10142-010-0162-7

[b71] Guo H., Ingolia N. T., Weissman J. S. & Bartel D. P. Mammalian microRNAs predominantly act to decrease target mRNA levels. Nature 466, 835–840 (2010). 20703300 10.1038/nature09267 PMC2990499

[b72] Koutsogiannaki S. & Kaloyianni M. Signaling molecules involved in immune responses in mussels. Inv. Surv. J. 7, 11–21 (2010).

[b73] Adema C. M. *et al.* Differential transcriptomic responses of *Biomphalaria glabrata* (Gastropoda, Mollusca) to bacteria and metazoan parasites, *Schistosoma mansoni* and *Echinostoma paraensei* (Digenea, Platyhelminthes). Mol. Immunol. 47, 849–860 (2010). 19962194 10.1016/j.molimm.2009.10.019 PMC2814977

[b74] Morga B., Renault T., Faury N. & Arzul I. New insights in flat oyster *Ostrea edulis* resistance against the parasite *Bonamia ostreae*. Fish Shellfish Immun 32, 958–968 (2012). 10.1016/j.fsi.2012.01.026 22406616

[b75] Tang M. *et al.* Integrated analysis of miRNA and mRNA expression profiles in response to Cd exposure in rice seedlings. BMC Genomics 15, 835 (2014). 25273267 10.1186/1471-2164-15-835 PMC4193161

[b76] Prado-Alvarez M., Gestal C., Novoa B. & Figueras A. Differentially expressed genes of the carpet shell clam *Ruditapes decussatus* against *Perkinsus olseni*. Fish Shellfish Immun. 26, 72–83 (2009). 10.1016/j.fsi.2008.03.002 19028428

[b77] Buckley K. M. & Rast J. P. Diversity of animal immune receptors and the origins of recognition complexity in the deuterostomes. Dev. Comp. Immunol. 49, 179–189 (2015). 25450907 10.1016/j.dci.2014.10.013

[b78] Pei H. *et al.* Integrative analysis of miRNA and mRNA profiles in response to ethylene in rose petals during flower opening. PloS One 8, e64290 (2013). 23696879 10.1371/journal.pone.0064290 PMC3655976

[b79] Andrews S. FastQC: A quality control tool for high throughput sequence data. *Reference Source* (2010) Available at: http://www.bioinformatics.bbsrc.ac.uk/projects/fastqc/.

[b80] Benson D. A. *et al.* GenBank. Nucleic Acids Res. 41, D36–D42 (2013). 23193287 10.1093/nar/gks1195 PMC3531190

[b81] Griffiths-Jones S. *et al.* Rfam: annotating non-coding RNAs in complete genomes. Nucleic Acids Res. 33, D121–D124 (2005). 15608160 10.1093/nar/gki081 PMC540035

[b82] Langmead B. & Salzberg S. L. Fast gapped-read alignment with Bowtie 2. Nat. Methods 9, 357–359 (2012). 22388286 10.1038/nmeth.1923 PMC3322381

[b83] Kozomara A. & Griffiths-Jones S. miRBase: annotating high confidence microRNAs using deep sequencing data. Nucleic Acids Res. gkt1181 (2013). 10.1093/nar/gkt1181 PMC3965103 24275495

[b84] Friedländer M. R. *et al.* Discovering microRNAs from deep sequencing data using miRDeep. Nat. Biotechnol. 26, 407–415 (2008). 18392026 10.1038/nbt1394

[b85] Anders S. Analysing RNA-Seq data with the DESeq package. Mol. Biol. 1–17 (2010).

[b86] Robinson M. D., McCarthy D. J. & Smyth G. K. edgeR: a Bioconductor package for differential expression analysis of digital gene expression data. Bioinformatics 26, 139–140 (2010). 19910308 10.1093/bioinformatics/btp616 PMC2796818

[b87] Anders S. & Huber W. Differential expression analysis for sequence count data. Genome Biol. 11, R106 (2010). 20979621 10.1186/gb-2010-11-10-r106 PMC3218662

[b88] Trapnell C. *et al.* Differential analysis of gene regulation at transcript resolution with RNA-seq. Nat. Biotechnol. 31, 46–53 (2013). 23222703 10.1038/nbt.2450 PMC3869392

[b89] Conesa A. *et al.* Blast2GO: a universal tool for annotation, visualization and analysis in functional genomics research. Bioinformatics 21, 3674–3676 (2005). 16081474 10.1093/bioinformatics/bti610

[b90] Ye J. *et al.* WEGO: a web tool for plotting GO annotations. Nucleic Acids Res. 34, W293–W297 (2006). 16845012 10.1093/nar/gkl031 PMC1538768

[b91] Kanehisa M. & Goto S. KEGG: kyoto encyclopedia of genes and genomes. Nucleic Acids Res. 28, 27–30 (2000). 10592173 10.1093/nar/28.1.27 PMC102409

[b92] Beißbarth T. & Speed T. P. GOstat: find statistically overrepresented Gene Ontologies within a group of genes. Bioinformatics 20, 1464–1465 (2004). 14962934 10.1093/bioinformatics/bth088

[b93] Morgan M., Falcon S. & Gentleman R. GSEABase: Gene set enrichment data structures and methods. R package version 1 (2008).

