# Supplementary material for: High throughput sequencing of small RNAs transcriptomes in two Crassostrea oysters identifies microRNAs involved in osmotic stress response
Source: Sci Rep. 2016 Mar 4;6:22687. doi: 10.1038/srep22687 (PMC4778033; doi:10.1038/srep22687)
Supplement: Supplementary Information [file srep22687-s1.pdf]

High throughput sequencing of small RNAs transcriptomes in two *Crassostrea* oysters identifies microRNAs involved in osmotic stress response

Xuelin Zhao[1], Hong Yu[1], Lingfeng Kong[1], Shikai Liu[1,2], Qi Li[1*]

[1]*Key Laboratory of Mariculture, Ministry of Education, Ocean University of China, Qingdao 266003, China*

[2]*The Fish Molecular Genetics and Biotechnology Laboratory, School of Fisheries, Aquaculture, and Aquatic Sciences and Program of Cell and Molecular Biosciences, Auburn University, Auburn, AL 36849, USA*

[*]Corresponding author: Q. Li, Fax: +86-532-82031765; E-mail address: qili66@ouc.edu.cn

Xuelin Zhao (xlzhao1989@163.com)
Hong Yu (hongyu@ouc.edu.cn)
Lingfeng Kong (klfaly@ouc.edu.cn)
Shikai Liu (szl0021@ auburn.edu)
Qi Li (qili66@ouc.edu.cn)

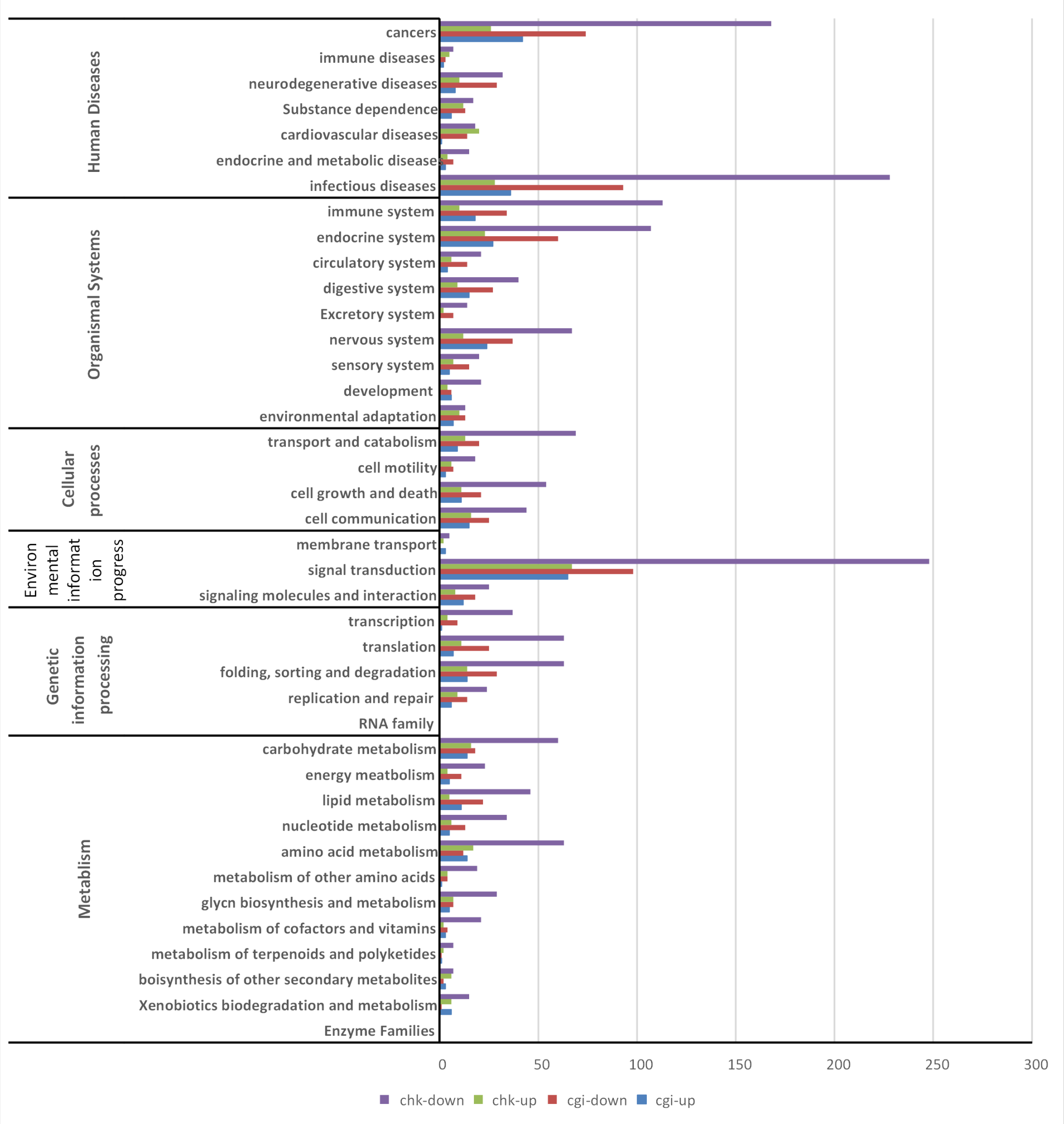

Figure S1. KEGG pathway distribution of target genes of differentially expressed miRNA of *C. gigas* and *C. hongkongensis*. Purple: target genes of down-regulated miRNAs in *C. hongkongensis*; Green: target genes of up-regulated miRNAs in *C. hongkongensis*; Red: target genes of down-regulated miRNAs in *C. gigas*; Blue: target genes of up-regulated miRNAs in *C. gigas*.
